# Germline breast cancer susceptibility genes, tumor characteristics, and survival

**DOI:** 10.1186/s13073-021-00978-9

**Published:** 2021-12-02

**Authors:** Peh Joo Ho, Alexis J. Khng, Hui Wen Loh, Weang-Kee Ho, Cheng Har Yip, Nur Aishah Mohd-Taib, Veronique Kiak Mien Tan, Benita Kiat-Tee Tan, Su-Ming Tan, Ern Yu Tan, Swee Ho Lim, Suniza Jamaris, Yirong Sim, Fuh Yong Wong, Joanne Ngeow, Elaine Hsuen Lim, Mei Chee Tai, Eldarina Azfar Wijaya, Soo Chin Lee, Ching Wan Chan, Shaik Ahmad Buhari, Patrick M. Y. Chan, Juliana J. C. Chen, Jaime Chin Mui Seah, Wai Peng Lee, Chi Wei Mok, Geok Hoon Lim, Evan Woo, Sung-Won Kim, Jong Won Lee, Min Hyuk Lee, Sue K. Park, Alison M. Dunning, Douglas F. Easton, Marjanka K. Schmidt, Soo-Hwang Teo, Jingmei Li, Mikael Hartman

**Affiliations:** 1 Genome Institute of Singapore, Human Genetics, Singapore, Singapore; 2 Saw Swee Hock School of Public Health, National University of Singapore and National University Health System, Singapore, Singapore; 3 School of Mathematical Sciences, Faculty of Science and Engineering, University of Nottingham Malaysia, Jalan Broga, 43500 Semenyih, Selangor Malaysia; 4 Cancer Research Malaysia, 1 Jalan SS12/1A, 47500 Subang Jaya, Selangor Malaysia; 5 Subang Jaya Medical Centre, Jalan SS 12/1A, 47500 Subang Jaya, Selangor Malaysia; 6 Department of Surgery, Faculty of Medicine, University of Malaya, Kuala Lumpur, Malaysia; 7 UM Cancer Research Institute, Kuala Lumpur, Malaysia; 8 Department of Breast Surgery, Singapore General Hospital, Singapore, Singapore; 9 Division of Surgical Oncology, National Cancer Centre Singapore, Singapore, Singapore; 10 Department of General Surgery, Sengkang General Hospital, Singapore, Singapore; 11 Division of Breast Surgery, Changi General Hospital, Singapore, Singapore; 12 Department of General Surgery, Tan Tock Seng Hospital, Singapore, 308433 Singapore; 13 Lee Kong Chian School of Medicine, Singapore, Singapore; 14 Institute of Molecular and Cell Biology, Singapore, Singapore; 15 KK Breast Department, KK Women’s and Children’s Hospital, Singapore, 229899 Singapore; 16 Division of Radiation Oncology, National Cancer Centre Singapore, Singapore, Singapore; 17 Lee Kong Chian School of Medicine, Nanyang Technology University, Singapore, Singapore; 18 Cancer Genetics Service, National Cancer Centre Singapore, Singapore, Singapore; 19 Oncology Academic Clinical Program, Duke NUS, Singapore, Singapore; 20 Division of Medical Oncology, National Cancer Centre Singapore, Singapore, Singapore; 21 Department of Hematology-oncology, National University Cancer Institute, National University Health System, Singapore, 119074 Singapore; 22 Department of Surgery, University Surgical Cluster, National University Hospital, Singapore, Singapore; 23 Department of Surgery, Breast Care Center, Daerim St. Mary’s Hospital, Seoul, Korea; 24 Department of Surgery, University of Ulsan College of Medicine and Asan Medical Center, Seoul, Republic of Korea; 25 Department of Surgery, Soonchunhyang University and Hospital, Seoul, Republic of Korea; 26 Department of Preventive Medicine, Seoul National University College of Medicine, Seoul, Republic of Korea; 27 Department of Biomedical Sciences, Seoul National University College of Medicine, Seoul, Republic of Korea; 28 Cancer Research Institute, Seoul National University, Seoul, Republic of Korea; 29 Centre for Cancer Genetic Epidemiology, Department of Oncology, University of Cambridge, Cambridge, UK; 30 Centre for Cancer Genetic Epidemiology, Department of Public Health and Primary Care, University of Cambridge, Cambridge, UK; 31 Division of Molecular Pathology, Netherlands Cancer Institute, Antoni van Leeuwenhoek Hospital, Amsterdam, the Netherlands; 32 Department of Surgery, Faculty of Medicine, University of Malaya, Jalan Universiti, 50630 Kuala Lumpur, Malaysia; 33 Department of Surgery, Yong Loo Lin School of Medicine, National University of Singapore and National University Health System, Singapore, Singapore

**Keywords:** Breast cancer, Protein-truncating variants, Overall survival

## Abstract

**Background:**

Mutations in certain genes are known to increase breast cancer risk. We study the relevance of rare protein-truncating variants (PTVs) that may result in loss-of-function in breast cancer susceptibility genes on tumor characteristics and survival in 8852 breast cancer patients of Asian descent.

**Methods:**

Gene panel sequencing was performed for 34 known or suspected breast cancer predisposition genes, of which nine genes (*ATM*, *BRCA1*, *BRCA2*, *CHEK2*, *PALB2*, *BARD1*, *RAD51C*, *RAD51D*, and *TP53*) were associated with breast cancer risk. Associations between PTV carriership in one or more genes and tumor characteristics were examined using multinomial logistic regression. Ten-year overall survival was estimated using Cox regression models in 6477 breast cancer patients after excluding older patients (≥75years) and stage 0 and IV disease.

**Results:**

PTV_9genes_ carriership (*n* = 690) was significantly associated (*p* < 0.001) with more aggressive tumor characteristics including high grade (poorly vs well-differentiated, odds ratio [95% confidence interval] 3.48 [2.35–5.17], moderately vs well-differentiated 2.33 [1.56–3.49]), as well as luminal B [HER−] and triple-negative subtypes (vs luminal A 2.15 [1.58–2.92] and 2.85 [2.17–3.73], respectively), adjusted for age at diagnosis, study, and ethnicity. Associations with grade and luminal B [HER2−] subtype remained significant after excluding *BRCA1/2* carriers. PTV_25genes_ carriership (*n* = 289, excluding carriers of the nine genes associated with breast cancer) was not associated with tumor characteristics. However, PTV_25genes_ carriership, but not PTV_9genes_ carriership, was suggested to be associated with worse 10-year overall survival (hazard ratio [*CI*] 1.63 [1.16–2.28]).

**Conclusions:**

PTV_9genes_ carriership is associated with more aggressive tumors. Variants in other genes might be associated with the survival of breast cancer patients. The finding that PTV carriership is not just associated with higher breast cancer risk, but also more severe and fatal forms of the disease, suggests that genetic testing has the potential to provide additional health information and help healthy individuals make screening decisions.

**Supplementary Information:**

The online version contains supplementary material available at 10.1186/s13073-021-00978-9.

## Background

Breast cancer is the most common cancer among women worldwide. Breast cancer manifestations are biologically and molecularly heterogeneous with a high degree of diversity observed between and within tumors. Such phenotypic differences in tumor characteristics are clinically informative because they are prognostic and can improve therapy selection [1, 2]. In particular, profiling of breast cancer can be based on the expressions of three immunohistochemical markers: estrogen receptor (ER), progesterone receptor (PR), and human epidermal growth factor receptor (HER2). Patients with ER- and PR-positive breast cancer respond well to endocrine therapy and have favorable outcomes [1]. In contrast, tumors which were ER/PR and HER2 negative are associated with worse survival and are typically treated with chemotherapy and radiotherapy [1].

There is a strong genetic component to the risk of breast cancer [3]. A large majority of disease-associated variants in susceptibility genes are protein-truncating variants (PTVs), a class of variants that usually results in an absence of functional protein [4]. Pathogenic PTVs are typically rare (allele frequency <1%) and are usually associated with a twofold or higher risk of breast cancer [5].

Previously, Li et al. [6] observed, in a study of 5099 Swedish breast cancer patients, that tumors arising in PTV carriers with known or suspected predisposition genes were phenotypically more aggressive and had worse survival as compared to tumors in non-carriers. However, there is growing concern that genetic markers identified in populations of predominantly European ancestry may not be equally informative in non-European populations, due to the modifier effect of lifestyle and genetic factors which may be distributed differently in diverse populations [7]. Here, we attempt to study how rare variants in breast cancer predisposition genes are associated with tumor characteristics and survival in 8852 breast cancer patients of Asian descent.

## Methods

### Study populations

The study population was derived from the patients enrolled in the Singapore Breast Cancer Cohort (SGBCC), the Malaysian Breast Cancer Genetic Study (MyBrCa), and the Korean Hereditary Breast Cancer Study (KOHBRA).

#### Singapore Breast Cancer Cohort (SGBCC)

The Singapore Breast Cancer Cohort (SGBCC) study includes women aged 21 years and above diagnosed with either breast carcinoma in situ or invasive breast cancer at one of the six participating restructured hospitals in Singapore (National University Hospital, KK Women’s and Children’s Hospital, Tan Tock Seng Hospital, Singapore General Hospital, National Cancer Centre Singapore, Changi General Hospital). These six hospitals collectively diagnose and treat ~76% of all breast cancer cases in Singapore [8]. Patients were a mixture of prevalent and incident cases from the three main ethnic groups, Chinese (81%), Malay (13%), and Indian (6%). Ethnicity was self-reported. According to the 2010 census of Singapore, Indian ethnicity refers “to persons of Indian, Pakistani, Bangladeshi or Sri Lankan origin such as Tamils, Malayalis, Punjabis, Bengalis, Singhalese, etc.” The ethnic distribution of SGBCC is similar to that of the general population of Singapore (Chinese 75.9%, Malay 15%, Indian 7.5%, Department of Statistics Singapore). Between April 2010 and December 2016, 7768 breast cancer patients were recruited into SGBCC. Of which 4538 patients had genetic information from blood or saliva samples collected at recruitment. After excluding duplicated individuals (*n* = 9), 4529 patients were included to test for associations between PTV carriership and tumor characteristics. A subset of 3213 patients were eligible for survival analysis.

#### The Malaysian Breast Cancer Genetic Study (MyBrCa)

The Malaysian Breast Cancer Genetic Study (MyBrCa), a hospital-based case-control study, was initiated in 2002 (described by Tan et al. [9]). Briefly, all patients diagnosed with breast cancer from two participating hospitals in Selangor, Malaysia (University Malaya Medical Centre, a public hospital, and Subang Jaya Medical Centre, a private hospital), were invited to participate. Participants were mainly from urban areas. These two hospitals treat more than 10% of the breast cancer cases in Malaysia [9]. The core ethnic groups of MyBrCa are Chinese (69%), Malay (16%), and Indian (12%). In Malaysia, the predominant ethnic group is Malay (67.4%), followed by Chinese (24.6%) and Indian (7.3%) (Department of Statistics Malaysia Official Portal). At recruitment, all participants (*n* = 3822) provided a blood or saliva sample and completed a detailed questionnaire that included lifestyle and reproductive-related factors for breast cancer as well as personal and family history of cancer. We excluded 46 related samples and 15 duplicated samples, resulting in 3761 patients included in the study of associations between PTV carriership and tumor characteristics. A subset of 3264 patients were eligible for survival analysis.

#### The Korean Hereditary Breast Cancer Study (KOHBRA)

The KOHBRA study is a prospective hereditary breast cancer cohort in Korea [10]. Between May 2007 and May 2010, the KOHBRA study recruited 1967 subjects from 35 hospitals registered in the Korean Breast Cancer Society. All participants received genetic counseling and *BRCA* genetic testing; the clinical information and blood samples for blood banking were collected. Included in the study were patients with a family history of breast or ovarian cancers, patients with non-familial breast and ovarian cancer but with other risk factors of genetic disease, and family members of breast cancer patients with *BRCA1/2* mutations [11]. Patients with information on PTVs (*n* = 562) were included in the study of associations between PTV carriership and tumor characteristics. The KOHBRA study was omitted from the survival analysis as it is a *BRCA1/2* case-control study oversampling *BRCA1/2* carriers, which will result in a survival bias in our PTV carriership analysis if included.

### Demographic information

Self-reported ethnicity (Chinese, Malay, Indian, and others) and family history of breast cancer (yes, no) were obtained from structured questionnaires, in SGBCC and MyBrCa. All patients from KOHBRA were Koreans and family history was obtained from a structured questionnaire.

### Clinical data for breast cancer patients

Clinical characteristics were extracted from hospital breast cancer registries or hospital medical records—age at diagnosis (years), tumor stage (0, I–IV), tumor size (<2 cm, 2–5 cm, and >5 cm, similar to TNM size reported by AJCC version 7), nodal status (positive, negative), tumor grade (well-differentiated, moderately differentiated, and poorly differentiated), and immunohistochemical markers ER, PR, and HER2. For ER status and PR status, staining of ≥1% was considered positive. HER2 status was classified as positive or negative (includes equivocal). Intrinsic-like subtypes were defined using immunohistochemical markers for ER, PR, and HER2 in conjunction with histologic grade: luminal A [ER+/PR+, HER2−, well- or moderately differentiated], luminal B [HER2−] (ER+/PR+, HER2−, and poorly differentiated), luminal B [HER2+] (ER+/PR+, HER2+, and poorly differentiated), HER2-enriched [HER2+], triple-negative [ER−, PR−, and HER2−] [12].

Treatment data, for SGBCC and MyBrCa, were extracted from hospital breast cancer registries or hospital medical records—surgery (yes, no), neo-adjuvant and/or adjuvant chemotherapy (yes, no), radiotherapy (yes, no), endocrine therapy (yes, no), and trastuzumab therapy (yes/no).

### Targeted sequencing

DNA isolation was performed according to the manufacturer’s instructions for buffy coat (FlexiGene DNA kit, Qiagen, or Promega’s Maxwell 16 Blood DNA Purification Kit) in SGBCC and MyBrCa, and G-DEX(TM) II Genomic DNA extraction kit (Intron) in KOHBRA. DNA isolation for saliva samples (only in SGBCC) was performed using Oragene and prepIT•L2P reagent, DNA Genotek.

Target-enriched sequencing libraries of germline DNA for the breast cancer cases and controls were prepared at the Centre for Cancer Genetic Epidemiology (University of Cambridge) as part of a larger effort (Breast Cancer Risk after Diagnostic Gene Sequencing, BRIDGES, https://bridges-research.eu) [13]. The gene panel, which was developed as part of the BRIDGES initiative, included coding sequences and intron/exon boundaries for a total of 34 genes for which there was prior evidence of association with breast cancer risk, including genes offered on commercial panels for breast cancer in early 2016 (Additional file 1: Table S1). The targeted sequencing workgroup in BRIDGES first performed rare variant detection in preliminary gene panels combined with data from whole-exome sequencing datasets before arriving at the 34 genes selected for the BRIDGES panel. Details of the library preparation, sequencing, variant calling, and quality control methods are given in Dorling et al. [13].

### Protein-truncating variant (PTV) carriership

In this study, PTVs were defined as (1) variants predicted to introduce a premature stop codon (frameshift or nonsense mutations), (2) small insertions or deletions (indels) predicted to disrupt a transcript’s reading frame, or (3) splice site mutations. PTVs occurring in the last exon of each gene were excluded to avoid including variants that do not lead to nonsense-mediated decay. Variants with less than 1% frequency in our study population were included. A list of PTVs from the 34 genes sequenced is listed in Additional file 1: Table S2. The distribution of PTVs in unselected breast cancer patients from SGBCC and MyBrCa was visualized in oncoplot and bar chart (Additional file 1: Fig. S1). As the PTVs are rare [14], the number of carriers in most genes was too small to analyze individually. Hence, we aggregated PTVs for each individual, creating a single binary variable: carrier of at least one PTV in any gene versus non-carrier. We hypothesized that the effects of PTVs on subtype and outcome were likely to be in the same direction and therefore the power to detect an association with this burden variable would be much greater.

### Statistical analysis

Fisher’s exact test was used to determine whether the proportion of PTV carriers for individual genes was different between two of the largest ethnic subgroups in this study (i.e., Chinese and Malay). To test for associations between PTV carriership and tumor characteristics in the full analytical cohort of 8852 breast cancer patients, we carried out multinomial logistic regression models (multinom function in the R package “nnet”) with tumor characteristics as the outcome, adjusting for age at diagnosis, study, and ethnicity. Odds ratios (ORs) and corresponding 95% confidence intervals (CIs) were estimated. While SGBCC and MyBrCa included three core ethnic groups, KOHBRA consisted of a homogeneous population of Koreans; hence, adjustment for study and ethnicity was done as a joint variable.

Overall survival was studied in a subset of patients (*n* = 6477, 73% of analytical cohort, KOHBRA study excluded). Additional file 1: Fig. S2 and Fig. S3 show the Kaplan-Meier curves including and excluding KOHBRA patients, respectively. Other exclusions made are as follows: (1) unknown age at diagnosis or age 75 and above at diagnosis [*n* = 240], (2) stage 0 [*n* = 711] or stage IV [*n* = 262] disease, (3) unknown recruitment or follow-up date [*n* = 10], and (4) patients with time at entry after the follow-up time of 10 years [*n* = 590]. Time at entry was defined as the time between the date of recruitment and the date of diagnosis. Follow-up time was defined as the time between the date of death/last known alive date and the diagnosis date, truncated at 10 years post-diagnosis.

Overall survival was studied using Cox proportional hazard models (survival package in R, where the *Surv(time at entry, follow-up time, event))* command was used to estimate hazard ratios (*HR*s) and corresponding *95% CI*. Adjustment for age at diagnosis, study (SGBCC, MyBrCa), ethnicity (Chinese, Malay, Indian, others), and tumor characteristics (stage, grade, ER status, nodal status, and tumor size) was done. As age 50 years is a common recommendation to start mammography screening, the survival analysis was repeated for subgroups of patients diagnosed at different age groups (<50 years and 50–75 years). A test for interaction (likelihood ratio test) between PTV and age group was performed using Cox proportional hazard models which included the following variables: PTV carriership, age group (<50, 50 to 75), study, ethnicity, and tumor characteristics.

### PTV gene subset analysis

In the recent study by Dorling et al. [13] involving 60,466 female breast cancer cases and 53,461 controls from 44 studies, PTVs in nine of the 34 genes (*ATM*, *BRCA1*, *BRCA2*, *CHEK2*, *PALB2*, *BARD1*, *RAD51C*, *RAD51D*, and *TP53*) were found to be strongly associated with breast cancer risk (Bayesian false-discovery probability, <0.05). Association and survival analyses were repeated for PTV carriership coded for these nine genes and separately for the remaining 25 genes. In the analysis of the remaining 25 genes, carriers of any of the nine genes associated with breast cancer risk were excluded.

### PTV subset analysis

As *BRCA1/2* carriers tend to be associated with more aggressive tumor characteristics [15], all analyses were repeated without *BRCA1/2* carriers to assess the combined effect of other breast cancer predisposition genes.

Intrinsic breast tumor subtypes are known to be highly predictive with breast cancer survival [16]. In addition, heterogeneity has been observed for breast cancer susceptibility risk genes across clinical subtypes for breast cancer [13, 17]. Hence, we performed further analysis to study the association of PTV carriership with tumor characteristics and survival was within each proxy subtype (luminal A, luminal B [HER2−], luminal B [HER2+], HER2-enriched [HER2+], and triple-negative).

As ethnic Chinese breast cancer patients comprise the majority of the study population (71%), all analyses were repeated on a subset of 6265 Chinese breast cancer patients.

All statistical analyses were performed using R version 4.0.2.

## Results

### Characteristics of the breast cancer cohorts

Table 1 summarizes the characteristics of the 8852 breast cancer patients included in this study. The median age at diagnosis was 51 years (interquartile range [*IQR*] 44 to 59). Incident cases made up 59% of all the breast cancer patients. The majority of the patients were Chinese (71%), followed by Malay (14%), Indian (8%), and Korean (6%). Fourteen percent of the patients reported having a family history of breast cancer. Additional file 1: Table S3 presents the characteristics of patients by study cohort.

**Table 1:** Characteristics of breast cancer patients. *IQR* interquartile range

	***N*** (%)
**Demographics**	
**Study**	
SGBCC	4529 (51%)
MyBrCa	3761 (42%)
KOHBRA	562 (6%)
**Case type**	
Incident	5229 (59%)
Prevalent	3587 (41%)
Unknown	36 (0%)
**Median age at diagnosis (IQR)**	51 (44–59)
*Unknown*	30
**Ethnicity**	
Chinese	6265 (71%)
Malay	1213 (14%)
Indian	707 (8%)
Korean	562 (6%)
Others	86 (1%)
Unknown	19 (0%)
**Family history**	
No	6968 (79%)
Yes	1220 (14%)
Unknown	664 (8%)
**Tumor characteristics**	
**Tumor behavior**	
In situ	1028 (12%)
Invasive	7478 (84%)
Unknown	346 (4%)
**Stage**	
0	759 (9%)
I	2244 (25%)
II	2746 (31%)
III	1123 (13%)
IV	270 (3%)
Unknown	1710 (19%)
**Nodal status**	
Negative	4765 (54%)
Positive	2889 (33%)
Unknown	1198 (14%)
**Tumor size, cm**	
≤2	1171 (13%)
2–5	1837 (21%)
>5	3916 (44%)
Unknown	1928 (22%)
**Grade**	
Well-differentiated	1029 (12%)
Moderately differentiated	3284 (37%)
Poorly differentiated	3038 (34%)
Unknown	1501 (17%)
**Estrogen receptor status**	
Positive	5542 (63%)
Negative	2242 (25%)
Unknown	1068 (12%)
**Progesterone receptor status**	
Positive	4722 (53%)
Negative	2820 (32%)
Unknown	1310 (15%)
**HER2 receptor status**	
Positive	1894 (21%)
Negative	4645 (52%)
Unknown	2313 (26%)
**Proxy subtype**	
Luminal A	2448 (28%)
Luminal B [HER2−]	837 (9%)
Luminal B [HER2+]	1033 (12%)
HER2-overexpressed	757 (9%)
Triple-negative	914 (10%)
Unknown	2863 (32%)
**Protein-truncating variants (PTVs)**	
**PTV 34 genes**	
Non-carrier	7873 (89%)
Carrier	979 (11%)
**PTV 9 genes**	
Non-carrier	8162 (92%)
Carrier	690 (8%)
**PTV 25 genes**	
Non-carrier	8524 (96%)
Carrier	328 (4%)

### PTV-associated tumors were phenotypically more aggressive

Approximately 11% of breast cancer patients (979 carriers) carried at least one PTV among the 34 genes sequenced (prevalence of 8% in Chinese, 9% in Malay, 9% in Indian, and 50% in Korean [KOHBRA was enriched for *BRCA1/2* carriers]) (Table 1). The proportion of PTV carriers in four genes were significantly different between Chinese and Malay breast cancer patients (Fisher’s exact test, *BRCA1*, *p* = 0.009; *BRCA2*, *p* = 0.008; *MUTYH*, *p* = 2.65E−4; *MSH6*, *p* = 0.003, Additional file 1: Table S4). The proportion of *BRCA1* carriers were approximately twofold higher in Malay breast cancer patients compared to Chinese breast cancer patients. *MUTYH* PTVs were more common in Malay patients while *MSH6* PTVs were more common in Chinese patients. However, multiple testing for 34 genes needs to be considered when interpreting these results.

Compared to tumors in breast cancer patients without any predicted PTV_34genes_ (adjusted for age at diagnosis, study, and ethnicity), tumors of PTV_34genes_ carriers were more likely to be of advanced stage (*OR*_stage II vs stage I_ 1.34 [1.10 to 1.63], *OR*_stage III vs stage I_ 1.29 [1.00 to 1.66]), more likely to be invasive (vs in situ, *OR* 1.48 [1.15 to 1.91]), node-positive (vs node-negative, *OR* 1.28 [1.10 to 1.49]), higher grade (poorly vs well-differentiated, *OR* 1.64 [1.26 to 2.14]), larger (*OR*_>5 vs ≤2cm_ 1.47 [1.11 to 1.93], *OR*_2–5 vs ≤2cm_ 1.39 [1.09 to 1.77]), ER-negative (vs ER-positive, *OR* 1.40 [1.19 to 1.64]), PR-negative (vs PR-positive, *OR* 1.40 [1.20 to 1.64]), and HER2-negative (vs HER2-positive, 1.42 [1.17 to 1.73]) (Table 2). In addition, PTV-associated tumors were more often of luminal B [HER2−] (vs luminal A, *OR* 1.68 [1.30 to 2.18]) and triple-negative subtypes (vs luminal A, *OR* 2.22 [1.76 to 2.80]). After the exclusion of 522 *BRCA1/2* carriers, stage (*OR*_stage II vs stage I_ 1.33 [1.03 to 1.72]), tumor size (*OR*_2–5cm vs ≤2cm_ 1.92 [1.33 to 2.77]), and subtype (*OR*_luminal B [HER2-] vs luminal A_ 1.46 [1.04 to 2.05]) remained significantly associated with PTV_34genes_ carriership (Table 2).

**Table 2:** Associations between protein-truncating variant [PTV] carriership (34 genes) and demographics, and tumor characteristics

	(i) PTV 34 genes				(ii) PTV 34 genes—excluding BRCA1/2 carriers			
	Non-carrier	Carrier	***OR*** (***95% CI***)	***p***	Non-carrier	Carrier	***OR*** (***95% CI***)	***p***
***Demographics***								
**Family history**								
No	6437	531	1.00 (reference)		6437	346	1.00 (reference)	
Yes	1061	159	**1.89 (1.56 to 2.29)**	**<0.001**	1061	81	**1.43 (1.12 to 1.84)**	**0.005**
Unknown	375	289	2.08 (1.27 to 3.41)	0.004	375	30	1.26 (0.61 to 2.58)	0.531
**Age group** (adjusted for ethnicity and study)								
≥50	4453	389	1.00 (reference)		4453	245	1.00 (reference)	
<50	3394	586	**1.36 (1.17 to 1.58)**	**<0.001**	3394	209	1.09 (0.90 to 1.32)	0.384
Missing age	26	4	2.61 (0.84 to 8.06)	0.096	26	3	3.23 (0.88 to 11.83)	0.077
**Ethnicity** (adjusted for age at diagnosis only)								
Chinese (MyBrCa)	2361	227	1.00 (reference)		2361	142	1.00 (reference)	
Chinese (SGBCC)	3390	287	0.91 (0.76 to 1.10)	0.341	3390	202	1.01 (0.81 to 1.26)	0.955
Malay (MyBrCa)	556	63	1.07 (0.79 to 1.44)	0.676	556	25	0.71 (0.45 to 1.10)	0.124
Malay (SGBCC)	542	52	1.00 (0.73 to 1.37)	0.989	542	31	0.95 (0.64 to 1.42)	0.818
Indian (MyBrCa)	404	48	1.25 (0.90 to 1.74)	0.185	404	26	1.08 (0.70 to 1.66)	0.729
Indian (SGBCC)	238	17	0.78 (0.47 to 1.30)	0.338	238	4	**0.28 (0.10 to 0.78)**	**0.014**
Korean	282	280	**8.38 (6.68 to 10.50)**	**<0.001**	282	24	1.28 (0.80 to 2.04)	0.308
Others/unknown	100	5	0.51 (0.18 to 1.39)	0.187	100	3	0.40 (0.10 to 1.63)	0.199
***Tumor characteristics***								
**Stage**								
0	703	56	0.83 (0.60 to 1.14)	0.247	703	34	0.96 (0.64 to 1.43)	0.822
I	2038	206	1.00 (reference)		2038	103	1.00 (reference)	
II	2421	325	**1.34 (1.10 to 1.63)**	**0.003**	2421	161	**1.33 (1.03 to 1.72)**	**0.028**
III	1007	116	**1.29 (1.00 to 1.66)**	**0.047**	1007	61	1.23 (0.89 to 1.70)	0.216
IV	240	30	1.49 (0.98 to 2.26)	0.064	240	11	0.96 (0.50 to 1.81)	0.889
Unknown	1464	246	**1.30 (1.05 to 1.61)**	**0.015**	1464	87	1.14 (0.85 to 1.54)	0.379
*Cumulative logistic model*	*6409*	*733*	***1.32 (1.14 to 1.52)***	***<0.001***	*6409*	*370*	*1.18 (0.98 to 1.42)*	*0.090*
**Tumor behavior**								
In situ	949	79	1.00 (reference)		949	43	1.00 (reference)	
Invasive	6628	850	**1.48 (1.15 to 1.91)**	**0.003**	6628	401	1.35 (0.97 to 1.87)	0.073
Unknown	296	50	1.13 (0.74 to 1.72)	0.583	296	13	0.74 (0.36 to 1.50)	0.401
**Nodal status**								
Negative	4282	483	1.00 (reference)		4282	240	1.00 (reference)	
Positive	2519	370	**1.28 (1.10 to 1.49)**	**0.002**	2519	168	1.20 (0.98 to 1.47)	0.081
Unknown	1072	126	0.89 (0.71 to 1.11)	0.290	1072	49	0.78 (0.56 to 1.07)	0.127
**Grade**								
Well-differentiated	947	82	1.00 (reference)		947	58	1.00 (reference)	
Moderately differentiated	2980	304	1.23 (0.94 to 1.61)	0.131	2980	154	0.85 (0.62 to 1.16)	0.311
Poorly differentiated	2672	366	**1.64 (1.26 to 2.14)**	**<0.001**	2672	177	1.09 (0.80 to 1.48)	0.577
Unknown	1274	227	1.25 (0.94 to 1.68)	0.124	1274	68	0.81 (0.56 to 1.18)	0.272
*Cumulative logistic model*	*6599*	*752*	***1.41 (1.21 to 1.64)***	***<0.001***	*6599*	*389*	*1.16 (0.95 to 1.41)*	*0.140*
**Tumor size, cm**								
≤2	1019	152	1.00 (reference)		1019	41	1.00 (reference)	
2–5	1599	238	**1.39 (1.09 to 1.77)**	**0.007**	1599	119	**1.92 (1.33 to 2.77)**	**<0.001**
>5	3582	334	**1.47 (1.11 to 1.93)**	**0.007**	3582	205	1.48 (0.98 to 2.23)	0.060
Unknown	1673	255	1.36 (1.06 to 1.74)	0.014	1673	92	1.38 (0.93 to 2.05)	0.108
*Cumulative logistic model*	*6200*	*724*	***1.24 (1.02 to 1.50)***	***0.030***	*6200*	*365*	*1.20 (0.92 to 1.55)*	*0.177*
**Estrogen receptor status**								
Positive	5034	508	1.00 (reference)		5034	283	1.00 (reference)	
Negative	1933	309	**1.40 (1.19 to 1.64)**	**<0.001**	1933	121	1.11 (0.89 to 1.38)	0.368
Unknown	906	162	1.20 (0.97 to 1.49)	0.092	906	53	0.96 (0.70 to 1.31)	0.786
**Progesterone receptor status**								
Positive	4296	426	1.00 (reference)		4296	232	1.00 (reference)	
Negative	2461	359	**1.40 (1.20 to 1.64)**	**<0.001**	2461	149	1.13 (0.91 to 1.40)	0.257
Unknown	1116	194	1.33 (1.09 to 1.62)	0.005	1116	76	1.20 (0.91 to 1.57)	0.200
**HER2 receptor status**								
Positive	1735	159	1.00 (reference)		1735	108	1.00 (reference)	
Negative	4061	584	**1.42 (1.17 to 1.73)**	**<0.001**	4061	235	0.93 (0.73 to 1.18)	0.537
Unknown	2077	236	1.12 (0.90 to 1.40)	0.302	2077	114	0.85 (0.65 to 1.12)	0.254
**Proxy subtype**								
Luminal A	2244	204	1.00 (reference)		2244	113	1.00 (reference)	
Luminal B [HER2−]	729	108	**1.68 (1.30 to 2.18)**	**<0.001**	729	52	**1.46 (1.04 to 2.05)**	**0.029**
Luminal B [HER2+]	947	86	0.97 (0.74 to 1.27)	0.823	947	59	1.24 (0.90 to 1.72)	0.196
HER2-overexpressed	705	52	0.83 (0.60 to 1.15)	0.272	705	36	1.02 (0.69 to 1.50)	0.912
Triple-negative	727	187	**2.22 (1.76 to 2.80)**	**<0.001**	727	51	1.38 (0.98 to 1.95)	0.065
Unknown	2521	342	1.20 (0.98 to 1.45)	0.072	2521	146	1.12 (0.86 to 1.44)	0.400

Models are adjusted for age at diagnosis (years) and the joint variable of study and ethnicity. Odds ratios (*OR*s) and 95% confidence intervals (*CI*s) were estimated using multinomial logistic regression models with each tumor characteristic as the outcome and genetic factors as explanatory variables; patients with unknown values were excluded from the analysis. *p*-value from Wald test. Results associated with *p* < 0.05 are denoted in bold

Compared to PTV_34genes_ carriership, the observed associations with tumor characteristics were generally larger in effect size when PTV_9genes_ carriership (i.e., nine genes found to be significant in Dorling et al. [18]) was evaluated (Table 3). After the omission of *BRCA1/2* carriers from the analysis, stage (*OR*_stage II vs stage I_ [*95% CI*] 1.86 [1.20 to 2.87]), grade (*OR*_poorly vs well-differentiated_ [*95% CI*] 3.67 [1.77 to 7.62]), size (*OR*_>5cm vs ≤2cm_ 2.03 [1.02 to 4.04]), and subtype (*OR*_luminal B [HER2-] vs luminal A_ 2.37 [1.44 to 3.91] and *OR*_triple-negative vs luminal A_ 1.90 [1.12 to 3.23]) remained significantly associated with PTV_9genes_ carriership (Table 3).

**Table 3:** Associations between protein-truncating variant [PTV] carriership (9 and 25 genes) and tumor characteristics

	(i) 9 genes				(ii) 9 genes—excluding BRCA1/2 carriers				(iii) 25 genes			
	Non-carrier	Carrier	***OR*** (***95% CI***)	***p***	Non-carrier	Carrier	***OR*** (***95% CI***)	***p***	Non-carrier	Carrier	***OR*** (***95% CI***)	***p***
**Stage**												
0	727	32	0.75 (0.49 to 1.14)	0.182	727	10	0.99 (0.48 to 2.05)	0.987	703	24	0.94 (0.58 to 1.50)	0.79
I	2111	133	1.00 (reference)		2111	30	1.00 (reference)		2038	73	1.00 (reference)	
II	2516	230	**1.48 (1.17 to 1.88)**	**0.001**	2516	66	**1.86 (1.20 to 2.87)**	**0.006**	2421	95	1.11 (0.81 to 1.52)	0.504
III	1047	76	1.36 (1.00 to 1.86)	0.051	1047	21	1.41 (0.80 to 2.49)	0.23	1007	40	1.15 (0.78 to 1.71)	0.478
IV	248	22	**1.85 (1.13 to 3.02)**	**0.015**	248	3	0.89 (0.27 to 2.93)	0.844	240	8	0.98 (0.47 to 2.07)	0.961
Missing	1513	197	1.54 (1.19 to 1.98)	<0.001	1513	38	1.74 (1.06 to 2.84)	0.028	1464	49	0.91 (0.62 to 1.32)	0.61
*Cumulative logistic model*	6649	493	**1.43 (1.20 to 1.70)**	**<0.001**	6649	130	1.29 (0.95 to 1.75)	0.108	*6409*	*192*	*1.03 (0.79 to 1.33)*	*0.83*
**Tumor behavior**												
In situ	979	49	1.00 (reference)		979	13	1.00 (reference)		949	30	1.00 (reference)	
Invasive	6878	600	**1.64 (1.19 to 2.26)**	**0.003**	6878	151	1.62 (0.91 to 2.88)	0.102	6628	250	1.22 (0.83 to 1.81)	0.309
Missing	305	41	1.30 (0.79 to 2.12)	0.302	305	4	0.50 (0.11 to 2.26)	0.37	296	9	0.84 (0.38 to 1.86)	0.662
**Nodal status**												
Negative	4438	327	1.00 (reference)		4438	84	1.00 (reference)		4282	156	1.00 (reference)	
Positive	2623	266	**1.34 (1.12 to 1.61)**	**0.002**	2623	64	1.28 (0.92 to 1.79)	0.139	2519	104	1.15 (0.89 to 1.49)	0.283
Missing	1101	97	0.97 (0.75 to 1.27)	0.848	1101	20	0.89 (0.53 to 1.48)	0.643	1072	29	0.72 (0.48 to 1.09)	0.121
**Grade**												
Well-differentiated	997	32	1.00 (reference)		997	8	1.00 (reference)		947	50	1.00 (reference)	
Moderately differentiated	3080	204	**2.33 (1.56 to 3.49)**	**<0.001**	3080	54	2.15 (1.02 to 4.54)	0.045	2980	100	**0.65 (0.46 to 0.92)**	**0.015**
Poorly differentiated	2767	271	**3.48 (2.35 to 5.17)**	**<0.001**	2767	82	**3.67 (1.77 to 7.62)**	**<0.001**	2672	95	**0.69 (0.48 to 0.97)**	**0.036**
Missing	1318	183	2.42 (1.60 to 3.66)	<0.001	1318	24	2.05 (0.90 to 4.65)	0.087	1274	44	0.62 (0.41 to 0.95)	0.028
*Cumulative logistic model*	6844	507	**1.88 (1.56 to 2.26)**	**<0.001**	6844	144	**2.04 (1.48 to 2.82)**	**<0.001**	6599	193	0.89 (0.68 to 1.17)	0.411
**Tumor size, cm**												
≤2	1047	124	1.00 (reference)		1047	13	1.00 (reference)		1019	28	1.00 (reference)	
2–5	1677	160	1.19 (0.90 to 1.56)	0.229	1677	41	**2.03 (1.08 to 3.81)**	**0.028**	1599	78	**1.84 (1.18 to 2.86)**	**0.007**
>5	3712	204	**1.57 (1.14 to 2.17)**	**0.006**	3712	75	**2.03 (1.02 to 4.04)**	**0.044**	3582	130	1.23 (0.75 to 2.04)	0.415
Missing	1726	202	1.44 (1.10 to 1.90)	0.009	1726	39	2.02 (1.04 to 3.90)	0.037	1673	53	1.10 (0.68 to 1.80)	0.693
*Cumulative logistic model*	6436	488	**1.34 (1.07 to 1.69)**	**0.012**	6436	129	1.54 (1.00 to 2.37)	0.05	*6200*	*187*	*1.11 (0.78 to 1.58)*	*0.55*
**Estrogen receptor status**	5218	324	1.00 (reference)		5218	99	1.00 (reference)					
Positive	2003	239	**1.66 (1.37 to 2.00)**	**<0.001**	2003	51	1.34 (0.95 to 1.89)	0.095	5034	184	1.00 (reference)	
Negative	941	127	1.34 (1.04 to 1.72)	0.021	941	18	0.92 (0.54 to 1.58)	0.773	1933	70	0.98 (0.74 to 1.30)	0.908
Missing									906	35	0.97 (0.67 to 1.42)	0.895
**Progesterone receptor status**												
Positive	4448	274	1.00 (reference)		4448	80	1.00 (reference)		4296	152	1.00 (reference)	
Negative	2548	272	**1.64 (1.36 to 1.98)**	**<0.001**	2548	62	1.35 (0.96 to 1.89)	0.08	2461	87	1.01 (0.77 to 1.33)	0.924
Missing	1166	144	1.40 (1.11 to 1.78)	0.005	1166	26	1.16 (0.73 to 1.84)	0.535	1116	50	1.21 (0.87 to 1.68)	0.26
**HER2 receptor status**												
Positive	1812	82	1.00 (reference)		1812	31	1.00 (reference)		1735	77	1.00 (reference)	
Negative	4203	442	**2.12 (1.64 to 2.73)**	**<0.001**	4203	93	1.30 (0.86 to 1.96)	0.211	4061	142	0.78 (0.59 to 1.04)	0.093
Missing	2147	166	1.56 (1.17 to 2.08)	0.002	2147	44	1.18 (0.74 to 1.90)	0.486	2077	70	0.73 (0.52 to 1.02)	0.061
**Proxy subtype**												
Luminal A	2320	128	1.00 (reference)		2320	37	1.00 (reference)		2244	76	1.00 (reference)	
Luminal B [HER2−]	753	84	**2.15 (1.58 to 2.92)**	**<0.001**	753	28	**2.37 (1.44 to 3.91)**	**<0.001**	729	24	1.01 (0.63 to 1.61)	0.981
Luminal B [HER2+]	989	44	0.75 (0.52 to 1.09)	0.133	989	17	1.08 (0.61 to 1.93)	0.789	947	42	1.31 (0.89 to 1.92)	0.175
HER2-overexpressed	732	25	**0.63 (0.40 to 0.99)**	**0.046**	732	9	0.77 (0.37 to 1.60)	0.483	705	27	1.15 (0.73 to 1.80)	0.548
Triple-negative	755	159	**2.85 (2.17 to 3.73)**	**<0.001**	755	23	**1.90 (1.12 to 3.23)**	**0.018**	727	28	1.13 (0.72 to 1.76)	0.601
Unknown	2613	250	1.30 (1.02 to 1.64)	0.033	2613	54	1.27 (0.83 to 1.95)	0.27	2521	92	1.04 (0.76 to 1.42)	0.804

All models are adjusted for study, ethnicity, and age at diagnosis. Odds ratios (ORs) and 95% confidence intervals (CIs) were estimated using multinomial logistic regression models with each tumor characteristic as the outcome and genetic factors as explanatory variables. PTV_9 genes_ includes *ATM*, *BRCA1*, *BRCA2*, *CHEK2*, *PALB2*, *BARD1*, *RAD51C*, *RAD51D*, and *TP53*. PTV_25 genes_ includes *ABRAXIS1*, *AKT1*, *BABAM2*, *BRIP1*, *CDH1*, *EPCAM*, *FANCC*, *FANCM*, *GEN1*, *MEN1*, *MRE11A*, *MLH1*, *MSH2*, *MSH6*, *MUTYH*, *NBN*, *NF1*, *PIK3CA*, *PMS2*, *PTEN*, *RAD50*, *RECQL*, *RINT1*, *SKT11*, and *XRCC2*. *p*-value from Wald test. Test for trend of stage, grade, and tumor size was done using the cumulative logistic model, excluding patients with missing values. Results associated with *p* < 0.05 are denoted in bold

Similar to PTV_9genes_ carriership after the exclusion of *BRCA1/2* carriers, PTV_25genes_ carriership of the remaining 25 genes was not significantly associated with most tumor characteristics, with the exception of tumor size (*OR*_2–5cm vs ≤2cm_ 1.84 [1.18 to 2.86]) (Table 3). Grade was also significantly associated with PTV_25genes_ carriership, but in the opposite direction grade (*OR*_moderately vs well-differentiated_ [*95% CI*] 0.65 [0.46 to 0.92], *OR*_poorly vs well-differentiated_ [*95% CI*] 0.69 [0.48 to 0.97]).

We further conducted subset analyses to evaluate the relationship between PTV carriership and tumor characteristics within subgroups of patients with breast cancers of different proxy subtypes (Table S5). Among patients of luminal A subtype, PTV_34genes_ and PTV_9genes_ carriership were associated with stage (*p*_cumulative logistic regression_ = 0.010 and 0.008, respectively) and nodal status (*OR*_positive vs negative_ 1.79 [1.31 to 2.45] and 1.95 [1.30 to 2.92], respectively). Among patients of luminal B [HER2+] subtype, PTV_34genes_ and PTV_9genes_ carriership were associated with tumor size (*p*_cumulative logistic regression_ = 0.013 and 0.008, respectively).

### PTV carriership and overall survival within 10 years post-diagnosis

In 6477 invasive non-metastatic breast cancer patients aged <75 years at diagnosis from SGBCC and MyBrCa, a total of 790 deaths due to any cause occurred within 10 years after diagnosis with a median follow-up time of ~5.6 years (*IQR* 3.4 to 8.5). The 10-year overall survival rate was 75% (*95% CI* 74 to 77%). In these 6477 patients, associations between PTV carriership and tumor characteristics were found to be similar with those from the entire analytical cohort of 8852 patients (Additional file 1: Table S6). Population structure of the major ethnic groups in SGBCC and MyBrCa was largely similar; Additional file 1: Fig. S4 shows a principal component analysis plot of SGBCC and MyBrCa breast cancer patients, colored by study (SGBCC or MyBrCa) and denoted by ethnicity (Chinese, Malay, or Indian).

PTV_34genes_ was not found to be associated with 10-year overall survival (Additional file 1: Table S7, Fig. S5). The results were not appreciably different after adjustment for age at diagnosis, study, ethnicity, stage, grade, ER status, nodal status, and tumor size or when *BRCA1/2* carriers were excluded (Additional file 1: Table S7, Table 4, and Additional file 1: Fig. S6).

**Table 4:** Associations between protein-truncating variant [PTV] carriership (34, 9, 25 genes) and overall survival

	(A) All ages				(B) <50 years				(C) 50 to 75 years			
	Alive	Dead	***HR*** (***95% CI***)	***p***	Alive	Dead	***HR*** (***95% CI***)	***p***	Alive	Dead	***HR*** (***95% CI***)	***p***
**(i) PTV 34 genes**												
Non-carrier	5213	710	1.00 (reference)		2152	308	1.00 (reference)		3061	402	1.00 (reference)	
Carrier	474	80	1.03 (0.81 to 1.29)	0.831	232	46	1.07 (0.78 to 1.46)	0.677	242	34	0.92 (0.65 to 1.31)	0.661
**(ii) PTV 34 genes (excluding** ***BRCA1/2*** **carriers)**												
Non-carrier	5213	710	1.00 (reference)		2152	308	1.00 (reference)		3061	402	1.00 (reference)	
Carrier	286	51	1.19 (0.89 to 1.58)	0.234	123	23	1.07 (0.69 to 1.64)	0.772	163	28	1.24 (0.84 to 1.82)	0.278
**(i) PTV 9 genes**												
Non-carrier	5386	746	1.00 (reference)		2229	324	1.00 (reference)		3157	422	1.00 (reference)	
Carrier	301	44	0.77 (0.57 to 1.05)	0.096	155	30	0.92 (0.63 to 1.34)	0.654	146	14	0.54 (0.32 to 0.92)	0.023
**(ii) PTV 9 genes (excluding** ***BRCA1/2*** **carriers)**												
Non-carrier	5386	746	1.00 (reference)		2229	324	1.00 (reference)		3157	422	1.00 (reference)	
Carrier	113	15	0.71 (0.42 to 1.18)	0.183	46	7	0.64 (0.30 to 1.36)	0.241	67	8	0.69 (0.34 to 1.40)	0.309
**(i)** ***BRCA1/2***												
Non-carrier	5499	761	1.00 (reference)		2275	331	1.00 (reference)		3224	430	1.00 (reference)	
Carrier	188	29	0.82 (0.56 to 1.19)	0.289	109	23	1.06 (0.69 to 1.63)	0.780	79	6	0.42 (0.19 to 0.94)	0.035
**(iii) PTV 25 genes (excluding carriers of PTV**_**9 genes**_**)**												
Non-carrier	5213	710	1.00 (reference)		2152	308	1.00 (reference)		3061	402	1.00 (reference)	
Carrier	173	36	1.63 (1.16 to 2.28)	0.004	77	16	1.48 (0.89 to 2.46)	0.131	96	20	1.77 (1.13 to 2.79)	0.013

All Cox proportional hazard models are adjusted for age at diagnosis (years), study (SGBCC or MyBrCa), ethnicity (Chinese, Malay, Indian, or others), stage (I, II, or III), grade (well-, moderately, or poorly differentiated), estrogen receptor status (positive, negative), nodal status (positive, negative), and tumor size (≤2, 2–5, or >5cm). (A) All ages and subgroups of (B) age at diagnosis <50 and (C) age at diagnosis 50 to 75. Patients considered in each association studied are indicated as follows: (i) all breast cancer patients, (ii) excluding *BRCA* carriers, and (iii) excluding carriers of PTV_9 genes_ (*ATM*, *BRCA1*, *BRCA2*, *CHEK2*, *PALB2*, *BARD1*, *RAD51C*, *RAD51D*, and *TP53*). Hazard ratios (*HR*s) and corresponding 95% confidence intervals (*CI*s) are shown. *SD* standard deviation

PTV_9genes_ carriership was not significantly associated with 10-year overall survival (Additional file 1: Table S7 and Fig. S7). After adjusting for age at diagnosis, study, ethnicity, stage, grade, ER status, nodal status, and tumor size, PTV_9genes_ carriership (*HR* 0.54 [0.32 to 0.92]) was associated with better overall survival in older breast cancer patients (50 to 75 years) (Table 4). Among younger patients, the *HR* (*95% CI*) was 0.92 (0.63 to 1.34) (*p* = 0.654). The protective effect among older patients remained significant when only *BRCA1/2* PTVs were considered in the gene subset (*HR* 0.42 [0.19 to 0.94], *p* = 0.035).

In contrast, PTV_25genes_ carriership was significantly associated with 10-year overall survival in the analysis including breast cancer patients of any age (adjusted *HR* 1.63 [1.16 to 2.28], Table 4). The effect size was larger for older breast cancer patients (adjusted *HR* 1.77 [1.13 to 2.79]) compared to younger patients (adjusted *HR* 1.48 [0.89 to 2.46], *p* = 0.131, Table 4 and Additional file 1: Fig. S8). The likelihood ratio tests (LRT) for interaction between PTV and age group (<50 years, 50 to 75 years) in the adjusted models (study, ethnicity, stage, grade, ER status, nodal status, and tumor size) were not statistically significant (*p*-values, PTV_34genes_ 0.507, PTV_9genes_ 0.084, and PTV_25genes_ 0.556). Further adjustments for treatment variables for all survival analyses did not appreciably change the results (Additional file 1: Table S9).

Kaplan-Meier curves for 10-year overall survival within each proxy subtype are presented in Additional file 1: Fig. S9 to S11. Significant associations were observed between PTV_34genes_ and PTV_25genes_ carriership and worse 10-year overall survival in the HER2-overexpressed proxy subtype. However, it should be noted that the results have to be interpreted with caution due to the limited number of death events within each subgroup.

### Subset analysis in Chinese breast cancer patients

The results observed for Chinese breast cancer patients were similar to that of all breast cancer patients, for associations between PTV carriership and tumor characteristics and also associations between PTV carriership and survival. While the *p*-values were no longer statistically significant due to the reduced sample size comprising only Chinese breast cancer patients, the observed odds ratios and hazard ratios were not appreciably different. These results are presented in Additional file 1: Tables S9 and S10.

## Discussion

Evaluating disease associations for rare variants in standard single-variant association analysis is challenging, since even if the effect size is large, the statistical power may still be low [19]. However, grouping variants in multimarker tests may substantially improve power [19]. For example, multiple common variants are frequently collapsed into a single polygenic risk score [20]. For rare variant analysis in a gene-based burden test, the number of individuals carrying variants in a given gene is compared between affected and unaffected groups [21]. While rare variant analyses are commonly carried out in a region or gene-based manner [13, 17], the collective effect of pathogenic variants across multiple breast cancer genes may also be studied [6, 22, 23]. In this large study of Asian breast cancer patients, we showed that PTV carriership in certain known or suggested breast cancer predisposition genes was associated with disease severity and fatality.

Two smaller studies on Asian Chinese breast cancer patients showed that while *BRCA1/2* PTV carriers were associated with more aggressive clinical features, PTV carriers of non-*BRCA1/2* breast cancer susceptibility genes when treated as a single group were not significantly associated with tumor characteristics (Wang et al., 480 patients, 65 PTV carriers for 20 genes [23]; Li et al., 936 patients, 223 PTV carriers for 40 genes [22]). However, in spite of differences in population ancestry, results from our large study of Asian breast cancer patients closely replicated the findings of a study comprising 5099 breast cancer patients of European descent [6]. The European study examined PTVs in 31 genes in an earlier version of the BRIDGES panel, of which 30 genes overlap with the 34 genes in our study. The overlap between the genes included in Li et al. [6] and the current study is shown in Additional file 1: Fig. S12. Li et al. [6] reported that compared to non-carriers, PTV carriers were more likely to have more aggressive tumors (i.e., ER-negative, large size, high grade, highly proliferative, luminal B, and triple-negative subtype). We observed the same significant associations in our Asian study and additionally found significant associations between PTV carriership and disease stage, tumor behavior, nodal involvement, PR status, and HER2 status.

Taking into account the known associations between *BRCA1/2*-related tumors and worse tumor biology, all analyses were repeated excluding *BRCA1/2* carriers. Li et al. [6] observed that PTV carriership remained associated with high grade and worse survival. In our study, a significant association with grade was no longer seen after *BRCA1/2* carrier exclusion, but other tumor characteristics common of fast-growing tumors, such as stage, tumor size, and luminal B [HER2−] subtype, remained significant.

A more recent study reported that not all of the 34 reported or known breast cancer susceptibility genes on the targeted sequencing panel were clinically relevant for the prediction of breast cancer risk [13]. The results of a subset analysis of the nine genes found to be associated with breast cancer risk suggest that the genes most relevant for breast cancer development were also associated with more aggressive tumor phenotypes (larger effect sizes observed than for 34 genes). Nonetheless, the worse tumor characteristics were mostly driven by *BRCA1/2* carriers—only stage, size, and subtype remained significantly associated with PTV carriership of the seven genes after the exclusion of *BRCA1/2* carriers. PTV carriership of the remaining 25 genes was associated with a larger tumor size. What we found to be different from the Li et al. [6] Swedish study was that in our study, among *BRCA1/2* non-carriers, there was a null relationship between PTV carriership (34 genes) and tumor grade. However, the gene subset analyses showed that while PTV_9genes_ carriership predisposed patients to worse tumor grade, a larger proportion of PTV_25genes_ carriers developed lower grade tumors.

We did not find a similar association between PTV_34genes_ carriership and 10-year overall survival as reported in Li et al.’s work on a Swedish dataset of 5099 breast cancer patients [6]. Interestingly, the gene subset analyses showed that PTV carriership of the nine genes, found to be most relevant for breast cancer risk, did not appear to be associated with worse survival. In particular, *BRCA1/2* conferred a survival benefit for patients diagnosed above 50 years of age. PTV carriership of the remaining 25 genes, however, increased the risk of dying from any cause in all age groups. This observation is unexpected as while PTV_25genes_ carriership was associated with larger tumor size, the tumors were of a lower grade. A possible explanation could be that these 25 genes are associated with other cancers, for which we do not have information on to perform a competing risk analysis. An alternative explanation could be that some genes are important drivers of certain tumor subtypes associated with worse survival, which we do not have the statistical power to study. Further work in a larger study population, perhaps involving sub-analyses by further division of PTV_25genes_ carriership, would be helpful in understanding this observation. Nonetheless, the results suggest that PTVs in breast cancer genes influence survival. Larger studies with higher statistical power will be needed to elucidate which of the genes are specifically associated with disease outcome.

The role of *BRCA1/2* in survival among breast cancer patients has been studied widely [24, 25]. Most studies found that germline *BRCA1/2* carriers were at a higher risk of death than their BRCA-negative counterpart, but there are indications that triple-negative breast cancer patients who are *BRCA1/2* carriers have a better prognosis [23, 24, 26]. As *BRCA1/2* carriership has the potential to affect the efficacy of chemotherapy, the effect on survival will need to be interpreted in light of the standard of care and patient population studied [26].

Among South-East Asians, it has been observed that Malay breast cancer patients tend to develop more aggressive tumors and have poorer survival rates [27]. The survival difference has been attributed to ethnic differences in lifestyle, socio-economic status, cultural values, tumor biology, and response to treatment [27]. In a previous study comparing Chinese and Malay breast cancer patients, a higher prevalence of *BRCA2* mutations was found among Malay breast cancer patients [28]. Our larger study supports this finding and found significant differences in the prevalence of some breast cancer genes (*BRCA1*, *MSH6*, and *MUTYH*) between the two ethnic groups, suggesting that germline genetics may impact ethnic differences in breast cancer tumor characteristics and survival. However, a larger study is needed to validate these ethnic differences.

There are limitations to this study. Although this study is the largest to date to examine the impact of rare variants on breast cancer tumor characteristics and survival among patients of Asian ancestry, the number of carriers is still too limited for individual gene evaluation. The numbers are also too small to make definitive conclusions in specific ethnic subgroups. The effect of large germline structural variants (e.g., deletions, duplications, insertions, inversions, and translocations), which are not limited to genetic changes in coding regions, was not evaluated in this study [29]. Such variants have been documented to contribute to 10–25% of pathogenic variants for hereditary disorders [29]. However, germline structural variants are less frequent in breast cancer genes such as *BRCA1* (0.8 to 6.9%) and *BRCA2* (5%) in Asian populations [30, 31]. As only germline variants were studied, we were not able to evaluate somatic second hits in tumors leading to the biallelic inactivation of the breast cancer genes. In view of potential bias, differences in the study design of each included cohort and how representative they are of the breast cancer demographics in general must be considered. For example, oversampling of *BRCA1/2* carriers in the KOHBRA study could have led to bias in the estimates, but limiting the analyses to two hospital-based cohorts (SGBCC and MyBrCa, unselected breast cancer patients) showed no evidence of such bias. Finally, we were not able to consider breast cancer-specific survival as the data was not consistently ascertained across all the studies. However, it is noteworthy that this large Asian breast cancer population studied is novel and timely in view of possible Eurocentric biases in genetic studies [7, 32].

## Conclusions

It is important to identify germline carriers of high-risk breast cancer PTVs to prioritize individuals for inclusion in cancer surveillance programs, which has the potential to save lives [33, 34]. The finding that PTV carriership is not just associated with higher breast cancer risk, but also more severe and fatal forms of the disease, suggests that genetic testing has the potential to provide additional health information and help healthy individuals make screening decisions.

## Supplementary Information

**Additional file 1.** Supplementary tables and figures. Description: This file contains Tables S1 to S10 and Fig. S1 to S12.

## Data Availability

Data from each study were previously described (SGBCC [8], MyBrCa [9], and KOHBRA [10]). The genetic and clinical data used in this study were obtained via a data request (concept #680) to the Breast Cancer Association Consortium (BCAC). All data requests can be directed to the BCAC data access committee (http://bcac.ccge.medschl.cam.ac.uk/bcacdata/).

## Abbreviations

*CI*: Confidence interval
ER: Estrogen receptor
HER2: Human epidermal growth factor receptor
*HR*: Hazard ratio
KOHBRA: Korean Hereditary Breast Cancer Study
MyBrCa: Malaysian Breast Cancer Genetic Study
*OR*: Odds ratio
PR: Progesterone receptor
PTV: Protein-truncating variant
SGBCC: Singapore Breast Cancer Cohort
